# Electronic Percolation Threshold of Self-Standing Ag-LaCoO_3_ Porous Electrodes for Practical Applications

**DOI:** 10.3390/ma12152359

**Published:** 2019-07-25

**Authors:** Stanica Enache, Mirela Dragan, Mihai Varlam, Konstantin Petrov

**Affiliations:** 1 National Research and Development Institute for Cryogenics and Isotopic Technologies—ICIT Rm. Vâlcea, 4th Uzinei Str., P.O. Box 7 Râureni, 240050 Vâlcea, Romania; 2 Acad. Evgeni Budevski Institute of Electrochemistry and Energy Systems, Bulgarian Academy of Sciences, Acad. G. Bonchev Str., Bl.10, 1113 Sofia, Bulgaria

**Keywords:** perovskites, sintering, electrical resistivity, composite media, effective medium theory

## Abstract

Perovskite LaCoO${}_{3}$ materials have various applications, from selective permeable membranes and gas sensing devices to water splitting applications. However, the intrinsic electrical resistivity of the perovskite limits the applicative potential. To overcome that, Ag powder was used with LaCoO${}_{3}$ to obtain porous composite electrodes with enhanced conductivities. For that, a series of composite Ag-LaCoO${}_{3}$ powders were prepared into pellets and pre-sintered at various temperatures up to 1000 ${}^{\circ }$C. Their structural properties and morphology were investigated by X-ray diffraction and scanning electron microscopy. The electronic transport of compacted specimens was studied by impedance spectroscopy. The results indicate that the presence of Ag acts as pre-sintering additive to obtain porous electrodes, with porosity values as high as 40% at 50 vol. % Ag. Moreover, the overall electrical resistivity of the composite electrodes varied well over four orders of magnitude. The results are discussed within the generalized Bruggeman theory for effective media comprising arbitrarily shaped metallic and semiconducting inclusions.

## 1. Introduction

Perovskite materials have opened the avenue of many modern research fields and discoveries, from high temperature superconductors and colossal magnetoresistance to ferroelectricity and spin dependent transport [1,2,3,4]. The fundamental aspects related to the interplay among their structural, magnetic and transport properties have generated huge potential applications and technological advances over the past decades [5,6,7]. The perovskites are commonly used as sensors and catalyst electrodes in fuel cells and are candidates for memory devices and spintronic applications [8,9,10,11]. In a search for alternative and renewable energy sources, perovskite materials are investigated as potential candidates for use in both photovoltaic and photocatalytic water splitting applications [12,13,14,15]. Moreover, Co-based perovskite materials are now considered for replacing platinum in catalytic converters in the automotive industry [16,17].

Among Co-based perovskites with potential application in clean energy harvesting, LaCoO${}_{3}$ has been shown to exhibit good electrochemical stability and catalytic activity towards the oxygen evolution reaction (OER) in alkaline media [18,19,20,21]. This makes it especially attractive to be used as anode material in electrolysis cells for medium and large scale hydrogen production stations, replacing more expensive catalysts based on ruthenium and iridium oxides [22,23,24]. For industrial applications, however, the production of large batches of LaCoO${}_{3}$ powders with specific crystalline quality [25], average particle size [26] and even grain shape [27] should be amenable upon demand. For that, one may choose between two distinct fabrication routes: dry processing (e.g., solid-state reaction [28] and ball-milling [29]) and wet synthesis (e.g., combustion [30], sol-gel [31], co-precipitation [32], hydrothermal [33], etc.). Both routes have advantages and disadvantages. For instance, wet synthesis is more laborious than dry processing since it often involves addition of chemical radicals to correct for fuel-to-oxidant ratio or adjusting the solution pH [34]. Although the wet synthesis methods lead to nm-size powders with uniform grain-size distribution [25,26], the dry processing methods are more direct, cost efficient and environmentally friendly. Additionally, dry processing does not make use of intermediate preparation steps or expensive chemicals other than the reaction precursors.

From an applicative point of view, the intrinsic properties of lanthanum cobaltite have to meet specific requirements in terms of crystalline quality, particle size and shape. For instance, for hydrogen production via water-splitting by alkaline electrolysis, powders of LaCoO${}_{3}$ with high specific area (i.e., >8 m${}^{2}$/g) are preferred [35,36,37] in order to enhance the catalytic activity towards oxygen evolution reaction [38,39]. Recent studies in the field have shown that nm-size LaCoO${}_{3}$ powders obtained by using a modified hydrothermal method exhibit high mass-specific OER activity of 7.51 A/g at 1.60 V vs. RHE [40]. This value is nearly four times higher than that of bulk LaCoO${}_{3}$ (i.e., 1.86 A/g). However, due to the sluggish proton-coupled electron transfer at the catalyst interface, further improvements of the OER kinetics require surface modification of the electrode–electrolyte interface [41]. One way to achieve that is by using metallic species that act as both conducting enhancers and surfactant binders. Among various metallic species, silver is a good candidate for several reasons: (i) Ag is stable in alkaline solutions, making it suitable to be used as electrode material; (ii) addition of Ag improves the overall electrical conductivity of composite Ag-LaCoO${}_{3}$ electrodes; and (iii) Ag grains act as local enhancers for the electronic transfer mechanism accompanying the oxygen evolution reaction in composite Ag-LaCoO${}_{3}$ electrodes [42,43]. Additionally, due to its low melting point (i.e., 960 ${}^{\circ }$C), Ag can be used as sintering additive for making self-standing conducting Ag-LaCoO${}_{3}$ electrodes by also keeping the porosity high.

In this work, we show that Ag-LaCoO${}_{3}$ composites with various contents of Ag-metal can be pelletized into self-standing electrodes by successive sintering in steps between 800 ${}^{\circ }$C and 1000 ${}^{\circ }$C for 1 h. The sintering process is accompanied by the formation of interconnected networks consisting of coalesced Ag grains throughout the entire sample. At a microscopic scale, the metallic Ag and perovskite LaCoO${}_{3}$ phases are well segregated, without forming intermediate compounds at grain boundaries. Upon sintering, the porosity of all electrodes remains high (i.e., over 40%) showing no significant dependence as a function of Ag content. On a macroscopic scale, however, the electrical transport properties are influenced dramatically. For pure LaCoO${}_{3}$, the sintering process promote grains to merge and coarse, leading to a decreased contact resistance at grain-boundaries. As a result of that, the overall electrical resistivity of LaCoO${}_{3}$ decreases by a factor 6, from 58 to 9.7 Ω m upon sintering at 800 ${}^{\circ }$C and 1000 ${}^{\circ }$C for 3 h, respectively. Comparatively, addition of Ag makes it possible to vary the electrical resistivity of compacted pellets by more than four orders of magnitude over a composition range less than 40 vol. % Ag. The results are discussed in the generalized framework for effective media consisting of metallic and semiconducting inclusions which also have an arbitrary shape with high geometrical symmetry (i.e., revolution ellipsoids or spheres). This approach is meaningful not only to understand the relation between the microstructure and the macroscopic physical properties of composite media, but it may also be useful to further manipulate the percolation threshold in composite electrodes with high porosity and enhanced electronic performance.

## 2. Experimental

### 2.1. Materials and Reagents

Lanthanum oxide (La${}_{2}$O${}_{3}$) (Sigma-Aldrich, 99.9%, Kandel, Germany), cobalt oxide (Co${}_{3}$O${}_{4}$) (Sigma-Aldrich, 99.7%, Kandel, Germany), silver (Ag) (Sigma-Aldrich, spherical, 120–325 mesh, 99.9%, Kendel, Germany) and isopropanol (C${}_{3}$H${}_{7}$OH) (Chimopar SA, >96%, Bucharest, Romania) were used without any further purification process.

### 2.2. Solid-State Synthesis of LaCoO${}_{3}$ Perovskite

LaCoO${}_{3}$ powders were obtained by solid-state reaction. For that, equimolar quantities of La${}_{2}$O${}_{3}$ and Co${}_{3}$O${}_{4}$ were mixed thoroughly in agate mortar using isopropanol as lubricant. The powder was then allowed to dry out and placed in alumina crucibles, moved to furnace and fired in air at 1000 ${}^{\circ }$C for 8 h, with a heating rate of 2 ${}^{\circ }$C/min.

### 2.3. Preparation of Composite Ag-LaCoO${}_{3}$ Mixtures

Up to 10 compositions were prepared by physically mixing 2 g of LaCoO${}_{3}$ (i.e., LCO) with 0.41*x* g of Ag powders (i.e., with *x* integer, from 1 to 10). Two grams of each composition were uniaxially pressed into a 2 cm in diameter pellet by using a stainless steel mould. All compositions were compacted under the same equivalent force value of 15 kg/cm${}^{2}$ (or 470 N). For comparison, pellets of pure Ag and LCO were also prepared. The compositions were labeled ALCO to denote mixtures of Ag and LaCoO${}_{3}$ powders. Each ALCO composition was indexed with *x*, from 1 to 10, according to the amount of Ag incorporated. ALCO0 and ALCO11 denote pure LaCoO${}_{3}$ and Ag, respectively.

### 2.4. Sintering of Composite Ag-LaCoO${}_{3}$ Pellets

ALCO pellets were sintered in steps of 50 ${}^{\circ }$C at temperatures from 800 ${}^{\circ }$C to 1000 ${}^{\circ }$C in air for 1 h, with a heating rate of 2 ${}^{\circ }$C/min. ALCO0 was sintered up to 1000 ${}^{\circ }$C for 3 h. ALCO11 was not heat treated. To avoid sample cross-contamination and to minimize Ag loss during sintering above the melting point of Ag (i.e., 960 ${}^{\circ }$C), the pellets were contained in closed alumina crucibles and heat-treated separately. Subsequently, the geometrical density of the pellets was determined from weight and size measurements. The brittle pellets, such as those with low Ag content, were moved to furnace for further sintering. Similarly, to avoid damaging the pellets, only the the robust ones were transferred to the impedance spectroscopy system for electrical conductivity measurements.

### 2.5. Characterization Techniques

All ALCO compositions were characterized by X-ray diffraction (XRD) using MiniFlex 600 Rigaku (Tokyo, Japan) with fixed Cu Kα source (λ = 0.1514 nm) and rotating silicon strip detector. 2θ scans were performed between 5${}^{\circ }$ and 90${}^{\circ }$, with a speed of 1 ${}^{\circ }$/min and resolution of 0.015 ${}^{\circ }$/step. The XRD peaks were indexed by using the Inorganic Crystal Structure Database (ICSD). Lattice constants and quantitative values for the identified phases were obtained from fit to the corresponding XRD spectra by using the PDXL powder diffraction analysis package from Rigaku.

Morphology was studied by using a Zeiss Field Emission Gun (FEG, Oberkochen, Germany) in conjunction with a scanning electron microscope (SEM). For that, the powder was dispersed in distilled water by using an ultrasonic bath and drop-cast on a conducting double faced carbon tape. Additionally, X-ray dispersive electron spectroscopy (EDX) was used to determine the stoichiometry of the perovskite phase.

Polished ALCO pellets with known thickness and diameter were mounted between two golden electrodes, which were firmly pushed against each other via a counter screw that was tightened in steps. This was repeated until the impedance values measured at 100 Hz leveled off. The holder containing the sample and a calibrated Pt-100 temperature sensor was mounted in the sample chamber of a flow cryostat system (from Novocontrol Technologies, Montabaur, Germany) in conjunction with a liquid nitrogen dewar and a transfer line in a feed-back loop with a temperature controller and a vacuum pump. The frequency dependent measurements were carried out at 25 ${}^{\circ }$C by using Alpha-A Performance Frequency Analyzer in the frequency domain between 10${}^{7}$ Hz and 10${}^{-2}$ Hz. Depending on sample characteristic (i.e., metal or semiconductor), the excitation amplitude was varied between 0.2 V and 2.0 V. All samples were heat-treated at 120 ${}^{\circ }$C to remove water, under continuous flow of nitrogen gas. During the precess, the impedance was monitored continuously until its value leveled off.

## 3. Results and Discussion

### 3.1. Structural and Morphological Characterization

**Figure 1:**
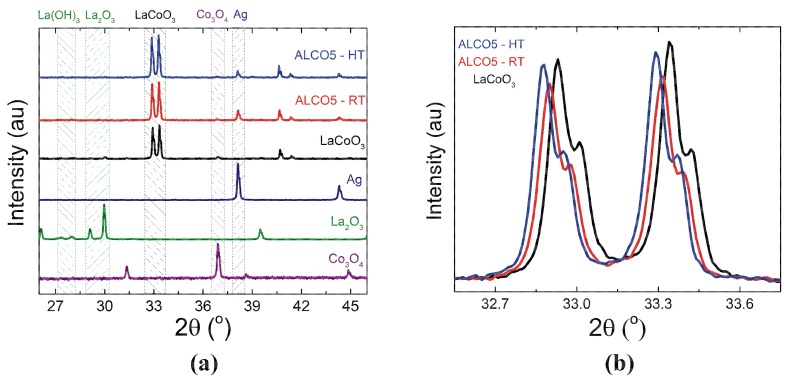
X-ray diffraction spectra of LaCoO${}_{3}$ and mixed Ag-LaCoO${}_{3}$ powders, with Ag to LaCoO${}_{3}$ weight ratio of 1 (sample ALCO5). (**a**) The diffraction patterns of LaCoO${}_{3}$ powders obtained from lanthanum and cobalt oxide precursors are illustrated, respectively. For comparison, ALCO5 powders mixed at room temperature (ALCO5-RT) and calcined at 1000 ${}^{\circ }$C for 1 h (ALCO5-HT) are shown. (**b**) The relative shift of the main Bragg reflections of the LaCoO${}_{3}$ phase in ALCO5-RT and ALCO5-HT powders is emphasized.

The crystal structure of the powders used in this work was investigated by X-ray diffraction. The results are shown in Figure 1. The main Bragg reflections corresponding to each constitutive phase is indicated, respectively.

In our previous work, we showed that single-phase perovskite LaCoO${}_{3}$ powders are readily obtained from lanthanum and cobalt oxide precursors by one-step solid-state synthesis at 1000 ${}^{\circ }$C for 8 h [44,45]. The crystal structure of LaCoO${}_{3}$ is rhombohedral (space group R−3c, ICSD 01-084-0845). The lattice constant values determined from fit to data are a=b=0.5443 nm and c=1.3094 nm. The promotor for the solid-state reaction is Co${}_{3}$O${}_{4}$, whose melting point (i.e., 850 ${}^{\circ }$ C) is much lower than that of La${}_{2}$O${}_{3}$ (i.e., 2315 ${}^{\circ }$C). La${}_{2}$O${}_{3}$ is partially segregated into La(OH)${}_{3}$. This is due to an enhanced affinity of La${}_{2}$O${}_{3}$ to decompose and form La(OH)${}_{3}$ in air. The stoichiometry of La${}_{2}$O${}_{3}$ can be recovered entirely, upon calcination at 600 ${}^{\circ }$C [46]. In Figure 1, the lattice constant values for hexagonal La(OH)${}_{3}$ (space group P63/m, ICSD 01-03-2034) are a=b=0.6529 nm and c=0.3859 nm, respectively. For tetragonal La${}_{2}$O${}_{3}$ (space group P−3m1, ICSD 01-071-5408), the corresponding lattice constants are a=b=0.3937 nm and c=0.6132 nm. Co${}_{3}$O${}_{4}$ is cubic (space group Fd3m, ICSD 01-074-1656), with lattice constant a=0.8083 nm.

The XRD spectra measured on mixed Ag-LaCoO${}_{3}$ powders exhibit all Bragg reflections of the constitutive phases. Compared to as-prepared LaCoO${}_{3}$ powders, the main XRD peaks corresponding to the perovskite LaCoO${}_{3}$ phase in Ag-LaCoO${}_{3}$ mix are shifted to slightly lower 2θ values. This feature is shown in Figure 1b for ALCO5 powders (i.e., with Ag to LaCoO${}_{3}$ weight ratio of 1) mixed at room temperature (ALCO5-RT) and heat-treated at 1000 ${}^{\circ }$C for 1 h (ALCO5-HT). From fit to data, the lattice constant values obtained for the perovskite LaCoO${}_{3}$ phase in ALCO5-RT powders are a=b=0.5448 nm and c=1.3094 nm, respectively. For ALCO5-HT, the corresponding lattice constants are a=b=0.5443 nm and c=1.3093 nm. Comparatively, the lattice constant value of Ag powder with cubic crystal structure (space group Fm3m, ICSD 01−071−4613) is a=0.4084 nm. The corresponding lattice constant values in ALCO5-RT and ALCO5-HT powders are a=0.4087 nm and a=0.4082 nm, respectively. Overall, the lattice constant values are similar within 0.5%. This indicates that no visible reaction occurred between Ag and LaCoO${}_{3}$, upon calcination at high temperatures.

**Figure 2:**
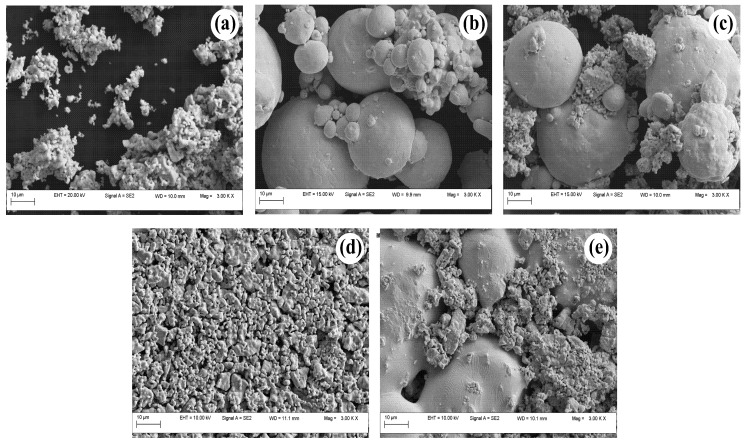
SEM images of Ag, LaCoO${}_{3}$ and mixed Ag-LaCoO${}_{3}$ powders and pre-sintered pellets: (**a**) perovskite LaCoO${}_{3}$ powders obtained by solid-state synthesis from lanthanum and cobalt oxide precursors at 1000 ${}^{\circ }$C for 8 h in air; (**b**) micrograph of the Ag powders used in this work; (**c**) mixed ALCO5 powders prepared at room temperature; and (**d**,**e**) micrographs of porous LaCoO${}_{3}$ and ALCO5 pellets sintered at 1000 ${}^{\circ }$C for 3 h and 1 h, respectively.

In Figure 2, we show the SEM images obtained on Ag, LaCo${}_{3}$ and Ag-LaCoO${}_{3}$ powders. The perovskite LaCoO${}_{3}$ powders synthesized by solid-state reaction at 1000 ${}^{\circ }$C for 8 h have micro-meter size and non-uniform particle-size distribution. They are clustered into pre-sintered conglomerates. EDX characterization indicates that the stoichiometry ratio of La:Co is essentially 1:1.

Compared to LaCoO${}_{3}$, Ag powders are spherical with much larger size and wider grain-size distribution. Smaller grains have tendency to group into conglomerates due to high surface energy. When physically mixed with LaCoO${}_{3}$ powders, Ag grains self-organize into interconnected networks with LaCoO${}_{3}$ filling the gap in-between. As a result of that, a composite medium comprising metallic Ag and semiconducting LaCoO${}_{3}$ phases forms. At grain-boundaries, physical contact is made between crystalline LaCoO${}_{3}$ grains and the surface of Ag, whose native state is Ag${}_{2}$O. The silver-oxide phase is superficial, below the detection limit of XRD. Above 280 ${}^{\circ }$C, Ag${}_{2}$O decomposes into elemental Ag. This explains the high stability of Ag towards formation of Ag doped oxides upon solid-state synthesis from Ag and oxide precursors. In Figure 2d, it is observed that Ag and LaCoO${}_{3}$ phases are well separated even after heat-treatment above the melting point of Ag. As a result of that, due to a high superficial tension of the molten phase, Ag grains coalesce and segregate into interconnected networks. From this perspective, it is expected that Ag would not influence much the sintering process rather than the overall conductivity of the sample, acting as a local enhancer for the electronic transport properties.

### 3.2. Pre-Sintering of Composite Ag-LaCoO${}_{3}$ Pellets

**Figure 3:**
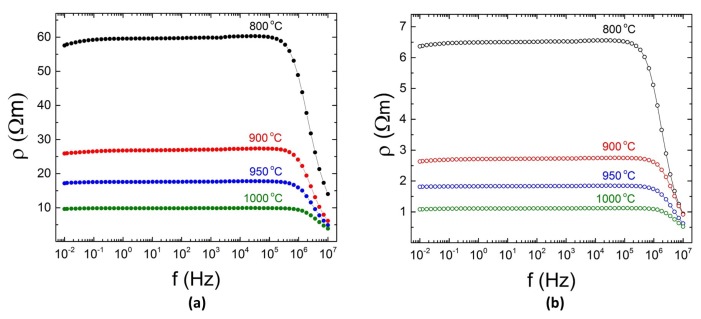
Frequency dependent impedance characterization of composite Ag-LaCoO${}_{3}$ pellets as a function of pre-sintering temperature. (**a**) The resistivities determined from measurements on LaCoO${}_{3}$ pre-sintered for 3 h at the given temperatures are shown. For comparison, the corresponding resistivities determined on ALCO5 pellets pre-sintered for 1 h are illustrated (**b**).

In Figure 3a, we show the electrical resistivity of pre-sintered LaCoO${}_{3}$ pellets determined from frequency dependent impedance characterization as a function of the pre-sintering temperature. All resistivity curves are essentially independent of frequency below 10${}^{5}$ Hz. The corresponding resistivity values are very high, typical for a semiconductor.

Upon sintering, interconnected LaCoO${}_{3}$ grains coalesce into randomly distributed networks that gradually merge into ideally dense structures through the entire sample. As a result of that, the contact resistance at grain-boundaries lowers, leading to an overall decrease of the electrical resistivity, as depicted in Figure 3. Addition of Ag, however, lowers the electrical resistivity by more than one order of magnitude, from 58 for LaCoO${}_{3}$ to 6.5 Ω m for ALCO5 sample heat-treated at 800 ${}^{\circ }$C. Further treatment at 1000 ${}^{\circ }$C leads to an even greater decrease in resistivity, from 9.7 for LaCoO${}_{3}$ to 1.1 Ω m for ALCO5. Compared to LaCoO${}_{3}$, ALCO5 sample is denser due to presence of Ag, whose grains also merge together upon heat-treatment, as shown in Figure 2e.

**Figure 4:**
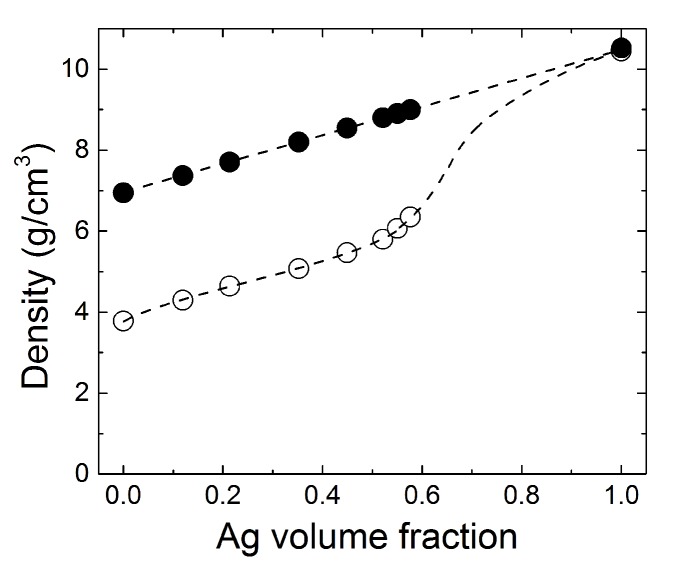
Theoretical (filled symbols) and experimental (open symbols) densities of composite Ag-LaCoO${}_{3}$ pellets. The theoretical density values were calculated for ideally dense pellets. The experimental densities were determined after heat-treatment at 1000 ${}^{\circ }$C. The dashed lines are guide to eye.

Compared to the theoretical density value of LaCoO${}_{3}$ (i.e., 6.94 g/cm${}^{3}$), the density determined for the LaCoO${}_{3}$ pellet pre-sintered at 1000 ${}^{\circ }$C for 3 h is 3.78 g/cm${}^{3}$. The pallet is nearly 46% porous and brittle. Addition of Ag makes it possible to obtain more compact pellets upon pre-sintering above the melting point of Ag. In Figure 4, we show the density values for the composite Ag-LaCoO${}_{3}$ pellets after heat-treatment at 1000 ${}^{\circ }$C. For comparison, the theoretical density values calculated for ideally 100% dense pellets are also shown. Both the theoretical and the experimental data fall on top of each other in samples with Ag compositions less than 50 vol. %. The porosity of these samples is almost 41%. Samples with higher volume of Ag are slightly denser, although the porosity remains above 37%.

To determine the theoretical density of composite Ag-LaCoO${}_{3}$ pellets as a function of the partial volume of Ag, the density values of LaCoO${}_{3}$ (i.e., 6.94 g/cm${}^{3}$) and Ag (i.e., 10.49 g/cm${}^{3}$) were used. In this work, we mixed 2 g of LaCoO${}_{3}$ with 0.41*x* grams of Ag, with *x* integer, with values from 0 to 10. For an ideally dense composite Ag-LaCoO${}_{3}$ pellet, the “effective” mass is: m${}^{*}\left(x\right)$ = m${}_{LCO}$ + m${}_{Ag}$ = (2 + 0.41*x*) grams. Correspondingly, the “effective” volume is: V${}^{*}\left(x\right)$ = V${}_{LCO}$ + V${}_{Ag}$ = (0.288 + 0.039*x*) cm${}^{3}$. From those, the composition dependent “effective" density is: m${}^{*}\left(x\right)$/V${}^{*}\left(x\right)$ = [(2 + 0.41*x*)/(0.288 + 0.039*x*)] g/cm${}^{3}$. With these given, the volume fraction of Ag-metallic inclusions in composite Ag-LaCoO${}_{3}$ pellets is *f* = V${}_{Ag}$/(V${}_{Ag}$ + V${}_{L}CO$) = 0.039x/(0.288+0.039x), where (1−f) represents the volume fraction of semiconducting LaCoO${}_{3}$.

### 3.3. Effective Medium Approach for Metal-Semiconductor Composite

Within the generalized medium theory derived by Bruggeman [47], the effective electrical resistivity ρ of a composite medium comprising metallic (M) and semiconducting (S) inclusions is described by the following equation:

(1) $$f\frac{\rho -\rho_{M}}{\rho +A\rho_{M}}+\left(1-f\right)\frac{\rho -\rho_{S}}{\rho +A\rho_{S}}=0$$

where *f* is the metallic volume fraction and $\rho_{M}$ and $\rho_{S}$ are the electrical resistivity values of metallic and semiconducting phases, respectively. In Equation (1), the shape of the constitutive inclusions is accounted by the geometrical factor *A*, whose value is related to the corresponding depolarization factor 0<D<1, e.g. A=1/D−1, with D=1/3 indicating spherical inclusions, while D>1.3 and D<1/3 correspond to prolate and oblate spheroids, respectively [48,49].

**Figure 5:**
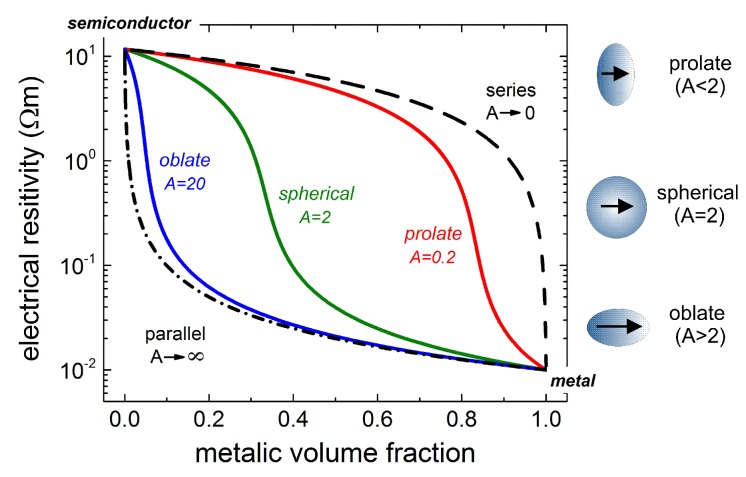
The effective electrical resistivity determined from Equation (1), according to the generalized Bruggeman theory for composite metal-semiconductor media with different values of the geometrical factor A, i.e., 2 for spherical, 0.2 for prolate and 20 for oblate inclusions. The curves corresponding to parallel and series resistors are also shown.

As a general comment on the values of *A* in Equation (1), the percolation threshold for the electrical resistivity in metal-semiconductor composites with spherical inclusions (i.e., with A=2) is exhibited at 33% metallic volume fraction. On a macroscopic scale, the electronic transport properties of such fully dense pellets are isotropic, although on a microscopic scale, the charge transport is driven through the lowest resistive path (i.e., metallic inclusions) mediated by percolation phenomena. On the other hand, oblate and prolate inclusions render the charge transport highly anisotropic. In such systems, the overall electrical resistivity is either metallic or semiconducting and it depends on the electrical field alignment with respect to the high symmetry axis of the constitutive spheroids. For instance, for extremely “deformed” oblate-spheroids (i.e., in Equation (1), the value of *A* is much higher than 2) with symmetry axis perpendicular on the electric field vector, the percolation threshold is shifted to lower values. In this case, the overall electrical resistivity is metallic-like resembling that of a mixed resistor system consisting of metallic and semiconducting sheets connected mainly in parallel. For prolate-spheroids (i.e., in Equation (1), the value of *A* is nearly 0), the percolation threshold is shifted to higher values so as the electrical resistivity is that of a semiconductor whose metallic and semiconducting inclusions are connected mainly in series. These features are illustrated in Figure 5, together with the corresponding shape of the constitutive inclusions in composite metal-semiconductor media. To calculate the electrical resistivity curves in Figure 5, the generic values of 1.7 Ω m for the semiconducting phase and 0.01 Ω m for the metallic one were used.

### 3.4. Electronic Transport in Composite Ag-LaCoO${}_{3}$ Media

**Figure 6:**
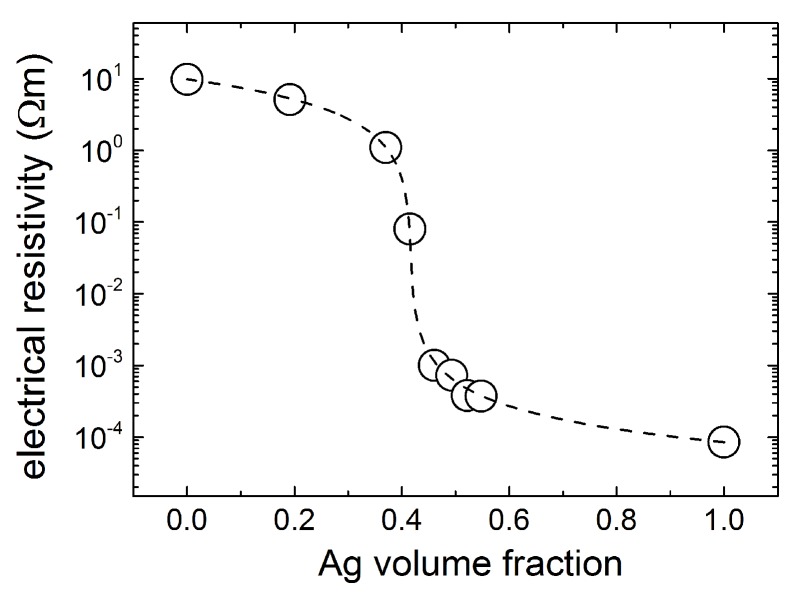
Electrical resistivity of composite Ag-LaCoO${}_{3}$ pellets sintered at 1000 ${}^{\circ }$C for 1 h. The dashed line is fit to data by using Equation (1). The obtained value of A≃1.5 corresponds to slightly deformed spheres.

In Figure 6, the electrical resistivity measured on composite Ag-LaCoO${}_{3}$ pellets is shown as a function of Ag volume fraction. Addition of Ag lowers the composite resistivity by almost five orders of magnitude, from 9.7 Ω m for LaCoO${}_{3}$ to 8.5 mΩ cm for compacted Ag powders. By using these values for the semiconducting and metallic electrical resistivities in Equation (1), a fit to the data in Figure 6 yields the value of 1.5 for the geometrical factor A. This value corresponds to slightly deformed spheres, with prolate geometry. The percolation threshold for the prolate inclusions with geometrical factor A≃1.5 is ∼41 vol. % Ag. This value is ∼8% higher than that for spherical inclusions (i.e., 33 vol. %).

The shift to higher values of the percolation threshold of the electrical resistivity measured on composite Ag-LaCoO${}_{3}$ media cannot be related to the relatively high porosity of the pellets. From the data in Figure 4, all pellets have essentially similar porosity below 50 vol. % Ag, i.e., above the percolation threshold for the electrical resistivity data in Figure 6. Therefore, it is more likely that the shift of the percolation threshold is rather correlated with the intrinsic morphology of the pellets, i.e., the metallic network created from merged Ag grains upon heat-treatment, as illustrated in Figure 2e.

Percolation threshold values higher or lower than 33 vol. % require more or less Ag to be used, respectively. For applications, it is, however, important to balance the electrode performance by keeping low both the material resources and the process costs. Therefore, it is, nevertheless, highly desirable to manipulate the percolation threshold toward lower values. This can be achieved either by increasing electrode porosity or by lowering the content of Ag. One way to do that is to use rod-shaped Ag powders instead of spherical. Alternatively, 3D Ag decorated powders might also be effective, although excessive grain coverage with Ag may hinder the catalytic activity of the perovskite material in the first place. In such circumstances, optimizing the electrode architecture for enhanced performance with minimal ohmic loss should be scrutinized beyond the effective medium theory, eventually.

## 4. Conclusions

In this work, we show that perovskite LaCoO${}_{3}$ powders were obtained directly by solid-state synthesis at 1000 ${}^{\circ }$C from lanthanum and cobalt oxide precursors, without making use of intermediate processing steps. The resulted powders are within a single phase with well defined grain-size distribution. Pellets of these powders are brittle and exhibit poor sintering behavior upon heat-treatment at 1000 ${}^{\circ }$C. Addition of Ag, however, makes it possible to obtained self-standing composite electrodes, which are also porous. Variation of the relative Ag volume fraction up to 50% does not change the porosity, whose value is ∼42%. On a microscopic scale, Ag and LaCoO${}_{3}$ do not form intermediate crystalline phases. The XRD data indicate that the cubic Ag and the perovskite LaCoO${}_{3}$ phases are well segregated upon treatment at high temperatures. At a macroscopic scale, however, addition of Ag influences dramatically the electrical resistivity of compacted pellets by more thanfour orders of magnitude, from 9.8 Ω m for pure LaCoO${}_{3}$ to ∼10${}^{-3}$Ω m for composite Ag-LaCoO${}_{3}$ with ∼50 vol. % Ag. The electrical resistivity data can be well described within an effective medium theory for composite media consisting of metallic and semiconducting inclusions with specific shape. That corresponds to slightly deformed spheres with prolate geometry. These findings are in reasonable good agreement with the physical shape of Ag grains, as shown by SEM.

Overall, the electronic transport properties of composite Ag-LaCoO${}_{3}$ media are assisted by percolative phenomena mediated by the least resistive path for electrons to follow. The percolation threshold value is exhibited at ∼41 vol. % Ag. Within the same framework, the percolation threshold of electrical resistivity is with 8% higher than that of composite media with spherical inclusions. To some extent, it is astonishing to see that the electrical resistivity data fall on a curve predicted by the effective medium theory, which is not that far from the ideal case. This is due to the tendency of Ag grains to coalesce at grain boundaries upon heat treatment. As a result of that, a metallic framework forms throughout the entire electrode, leading to an overall drop of the ohmic loss across the electrodes. Further ways to improve the overall performance of self-standing composite Ag-LaCoO${}_{3}$ electrodes aim at reducing the percolation threshold of electrical resistivity well below 33 vol. % Ag by also maintaining the porosity high.
